# Narcissism and coach interpersonal style: A self‐determination theory perspective

**DOI:** 10.1111/sms.12635

**Published:** 2015-12-21

**Authors:** D. Matosic, N. Ntoumanis, I. D. Boardley, C. Sedikides, B. D. Stewart, N. Chatzisarantis

**Affiliations:** ^1^ School of Sport Exercise & Rehabilitation Sciences University of Birmingham Birmingham UK; ^2^ School of Psychology & Speech Pathology Curtin University Perth Western Australia Australia; ^3^ Psychology Unit University of Southampton Southampton UK; ^4^ School of Psychology University of Birmingham Birmingham UK

**Keywords:** Controlling, autonomy‐supportive, dominance, empathy, sport

## Abstract

Athletes’ sport experiences are often influenced by the interpersonal styles of communication used by their coaches. Research on personality antecedents of such styles is scarce. We examined the link between a well‐researched personality trait, namely narcissism, and two types of coaching interpersonal style, namely autonomy‐supportive and controlling styles. We also tested the mediating roles of dominance and empathic concern in explaining the relations between narcissism and the two coaching interpersonal styles. United Kingdom‐based coaches (*N* = 211) from various sports completed a multi‐section questionnaire assessing the study variables. Regression analyses revealed a positive direct relation between narcissism and controlling coach behaviors. Furthermore, empathy (but not dominance) mediated the positive and negative indirect effects of narcissism on controlling and autonomy‐supported interpersonal styles, respectively. We discuss these findings in terms of their implications for coaching and the quality of athletes’ sport experiences.

Coaches’ behaviors can have a profound influence on their athletes’ motivation, performance, and well‐being (Ntoumanis & Mallet, 2014). Self‐Determination Theory (SDT; Ryan & Deci, 2002) provides an appropriate framework for investigating this topic, as it describes different (i.e., autonomy‐supportive and controlling) interpersonal styles (set of behaviors) relevant to coaching (Occhino et al., 2014). Much research has examined the consequences of these styles in sport (for a review, see Ntoumanis, 2012). As well as understanding the outcomes of different interpersonal styles, it is important to consider their potential antecedents. Research on the antecedents of these styles is limited and has neglected personality variables. In the current study, we investigate the role of one particular personality trait – narcissism – in predicting autonomy‐supportive and controlling coach behaviors.

Examples of autonomy‐supportive behaviors are offering meaningful choices, allowing volition or initiative, encouraging rationales for task engagement, and acknowledging negative feelings (Mageau & Vallerand, 2003). Considerable SDT‐based research points to positive relations between autonomy‐supportive behaviors and optimal (i.e., self‐determined) motivation (Ntoumanis & Standage, 2009), as well as other adaptive outcomes (e.g., well‐being; Bartholomew et al., 2011).

Conversely, controlling coach behaviors are evident when coaches are authoritarian and use pressuring techniques to impose specific ways of feeling, thinking, and behaving upon their athletes (Bartholomew et al., 2009). Controlling coaches use various manipulative strategies to influence their athletes, such as outcome‐contingent rewards (e.g., medals or money), imposed opinions, high‐handed statements, yelling, normative comparisons, and contingent affection (Bartholomew et al., 2009). SDT‐based research has shown positive relations between controlling behaviors and sub‐optimal (i.e., non‐self‐determined) motivation (Pelletier et al., 2001), as well as other maladaptive outcomes (e.g., ill‐being; Bartholomew et al., 2011).

To date, researchers have primarily focused on the outcomes of autonomy‐supportive and controlling behaviors, and much less on their antecedents. Mageau and Vallerand (2003) proposed a model of coach–athlete relationship, grounded in SDT, in which they specified three antecedent categories of coaching behaviors. Importantly, one of these categories is related to the coach's personal orientation. According to this model, personality traits or stable beliefs are parts of this personal orientation category and can influence the likelihood of a person adopting autonomy‐supportive vs controlling behaviors.

## Narcissism and coach interpersonal style

Narcissism, a well‐researched personality trait of leaders (Rosenthal & Pittinsky, 2006; Schoel et al., 2015), is relevant to the coaching literature, given the leading role of coaches in sport. Narcissism is a self‐centered, self‐aggrandizing, dominant, and manipulative interpersonal orientation (Emmons, 1987; Sedikides et al., 2004). Individuals high on narcissism (hereafter referred to as “narcissists” for brevity) seek attention and admiration, feel entitled, and are amoral, focusing on personal benefit, even at the blatant expense of others (Campbell et al., 2005; Morf et al., 2011; Watts et al., 2013). In group setting, narcissists emerge as leaders (due to their conversational dominance) and, more generally, strive to assume leadership positions (Brunell et al., 2008; Campbell et al., 2011).

Overall, the literature depicts narcissists negatively with regard to their leadership qualities and effectiveness (Judge et al., 2006; Grijalva et al., 2015a; Schoel et al., 2015). This is not surprising, given narcissists’ behaviors toward subordinates. Specifically, narcissists are constantly looking for validation (perhaps due to nagging feelings of insecurity; Gregg & Sedikides, 2010) and seek out situations through which they can assert their authority and superiority over others (Morf & Rhodewalt, 2001). Furthermore, they lack suitable cognitive and affective responding to others’ experiences (i.e., empathy; Davis, 1983), thus making self‐centered decisions that ignore suggestions from others (Rosenthal & Pittinsky, 2006). In summary, narcissistic leaders are driven by their own need for dominance and admiration without empathy for those whom they lead (Rosenthal & Pittinsky, 2006; Schoel et al., 2015).

The leadership qualities associated with narcissism suggest that this trait may be a potential explanatory antecedent of coach behaviors, particularly controlling behaviors, in sport. For example, narcissists often behave in an authoritarian manner, take advantage of others, are hypersensitive to criticism, and become hostile when their planned actions turn ineffective (Sedikides et al., 2002; Morf et al., 2011). They belittle others (Stucke, 2003) and aggress against critics of their sub‐par performance (Bushman & Baumeister, 1998). Belittlement and aggression are characteristics of the intimidation strategies associated with controlling coach behaviors (Bartholomew et al., 2009), consistent with the possibility that narcissistic coaches are more likely to enact controlling behaviors. Importantly, narcissists are attracted to highly competitive situations, because these provide them with the opportunity for self‐enhancement (Wallace & Baumeister, 2002). Similarly, controlling coaches value competition and focus mainly on winning as a measure of success (Bartholomew et al., 2009). Finally, narcissists regard themselves as responsible for team success, but blame team failure on others (Campbell et al., 2000). Comparably, controlling coaches employ strategies such as guilt‐inducing tactics to express their disappointment to seemingly underperforming athletes (Bartholomew et al., 2009). As such, it is reasonable to presume that narcissistic leaders in the sport coaching population exhibit controlling behaviors.

By comparison, very little is known about the relation between narcissism and autonomy‐supportive forms of behavior. Recent research on narcissism and prosociality has indicated that narcissism is negatively related to helping behaviors (Lannin et al., 2014). Helping is a benevolent act and could conceptually be aligned with some autonomy‐supportive behaviors such as providing rationales, offering encouragement, and being responsive to questions (Reeve & Jang, 2006). A situation in which narcissists might refuse to act prosocially is when helping others does not offer them the opportunity for self‐enhancement (Wallace & Baumeister, 2002). In such a situation, narcissistic coaches might opt against autonomy‐supportive strategies toward their athletes. However, when helping creates self‐enhancement opportunities, narcissists may engage in autonomy‐supportive behaviors.

## Mediators of the relation between coach narcissism and coach interpersonal style

The construct of empathy may be relevant as an explanation for the putative links between narcissism and coach interpersonal style. Lack of empathic concern accounts for the positive relation between narcissism and antisocial behavior (Miller & Eisenberg, 1988; Hepper et al., 2014a). More specifically, the affective component of empathy – termed empathic concern (i.e., the ability to share others’ emotions, feel sympathy, and experience compassion; Davis, 1980) – is often strongly and negatively associated with narcissism. As intimidation and additional controlling strategies enacted by coaches are characterized by aggression (Bartholomew et al., 2009), it is possible that reduced empathic concern in narcissistic coaches drives, in part, their controlling behaviors. Furthermore, as a form of “other‐oriented” empathy (Davis, 1983), empathic concern may be considered an ingredient of autonomy‐supportive behaviors (Soenens et al., 2007). On the basis of this literature, we hypothesized that empathic concern would mediate the relations between coach narcissism and coaching interpersonal style (i.e., controlling vs. autonomy‐supportive behaviors).

Another putative mediator of the proposed link between coach narcissism and coach interpersonal style is dominance. Dominance is the component of power (with the other components being status and authority; Keltner et al., 2003) that may have the potential to account best for relations between coach narcissism and controlling coaching behaviors. Dominance refers to the ability to direct subordinates by regulating their resources and establishing superiority over them (Sedikides et al., 2002; Keltner et al., 2003). Dominance is one of the most demonstrative features of narcissistic leaders, as it entails pressurizing, harassing, or intimidating displays. Controlling coaching behaviors aim to demonstrate superiority over others (Bartholomew et al., 2009), whereas autonomy‐supportive behaviors aim to support others, not dominate them. Hence, high dominance, a self‐centered orientation, may be associated with controlling behaviors, but not with autonomy‐supportive behaviors. On the basis of this literature, we tested whether dominance mediates the hypothesized relations between coach narcissism and controlling coaching behaviors.

## The current study

The primary purpose of this study was to examine the antecedent role of narcissism in predicting controlling vs autonomy‐supportive coach behaviors, in situations in which narcissism could be activated. On the basis of the above literature review, we hypothesized that coach narcissism would have a direct positive predictive effect on controlling coach behavior (Sedikides et al., 2002), and a direct negative predictive effect on autonomy‐supportive behavior (Lannin et al., 2014). In addition, we hypothesized that reduced empathic concern would mediate (a) a positive (indirect) effect of narcissism on controlling coach behavior (Hepper et al., 2014a), and (b) a negative (indirect effect) of narcissism on autonomy‐supportive coach behavior (Eisenberg et al., 2010). Finally, we hypothesized that dominance would mediate a positive (indirect) effect of narcissism on controlling coach behavior (Raskin et al., 1991).

## Method

### Participants

The sample included 211 professionally qualified coaches (178 male, 33 female; M_age_ = 38.30, SD = 14.16, range = 18–81 years old) from across the United Kingdom. They represented a variety (*n* = 28) of sports (e.g., football, rugby, cricket, swimming, athletics, tennis). We recruited coaches via the Sportscoach UK organisation, county partnerships, sports club websites, and social media (i.e., Twitter, LinkedIn). Participants had on average 13.51 (SD = 10.07) years of coaching experience and were mainly White British (89.10%).

### Measures

#### Autonomy‐supportive and controlling coach behaviors

We measured autonomy‐supportive and controlling coach behaviors as responses to 12 vignettes, available online as supplemental material. The vignettes corresponded to the 12 most important characteristics of narcissism: hypersensitivity to criticism, authority, self‐sufficiency, superiority, exhibitionism, exploitativeness, entitlement, feelings of inferiority, lack of empathy, amorality, arrogance, and grandiosity. The vignettes described common coaching situations that could evoke narcissistic characteristics in coaches. That is, the situations were intended to render salient a context in which coach narcissism would be active and relevant. For example, many of these situations represented a threat to the pertinent narcissistic characteristic, as the following vignette (referring to hypersensitivity to criticism) illustrates:

Upon the end of an important league game, the coach gathered his team on the field to discuss the team's defeat. After the coach finished talking, a team captain stood up criticizing the coach for the way the team played. The coach was visibly insulted and became intensely hostile in response to the criticism.

We asked coaches to rate what response would be appropriate in each vignette. The responses included examples of autonomy‐supportive behaviors (e.g., “Invite the player to a one‐on‐one meeting, to discuss how things might be resolved”) and controlling behaviors (e.g., “Shout to the player, threatening his captain's position”). Responses options ranged from 1 (*strongly disagree*) to 6 (*strongly agree*). We piloted extensively the vignettes and responses with coaches (*n* = 5) and SDT experts (*n* = 4), who provided feedback on the accuracy, content, and clarity of the vignettes and responses. We then made appropriate revisions.

#### Narcissism

We assessed narcissism via the 40‐item and forced‐choice Narcissistic Personality Inventory (NPI; Raskin & Terry, 1988). The NPI requires participants to choose between a narcissistic (e.g., “Modesty doesn't become me”) and a non‐narcissistic (e.g., “I am essentially a modest person”) statement. Scores range from 0 to 40, with higher scores reflecting higher narcissism.

#### Dominance

We assessed dominance using the 11‐item International Personality Item Pool Dominance Scale (Goldberg et al., 2006), which is based on the California Personality Inventory (Wink & Gough, 1990). Sample items are: “Put people under pressure” and “Impose my will on others.” Scores ranged from 1 (*strongly disagree*) to 6 (*strongly agree*).

#### Empathic concern

We assessed empathic concern with the 7‐item Empathic Concern Subscale of the Interpersonal Reactivity Scale (Davis, 1980). Sample items are: “When I see someone being treated unfairly, I sometimes don't feel very much pity for them” (reverse scored), and “Sometimes I don't feel sorry for other people when they are having problems” (reverse scored). Scores ranged from 0 (*does not describe me well*) to 4 (*describes me very well*).

### Procedures

Following university ethics approval, we created an online questionnaire using the Bristol Online Survey (BOS) platform. Coaches who consented to participate completed a multi‐section online (*n* = 210) or hardcopy (*n* = 6) questionnaire in 15–20 min.

### Data analyses

First, we used SPSS 21.0 to screen for univariate and multivariate normality (i.e., skewness and kurtosis), and for multicollinearity. We also calculated correlations, means, standard deviations, and scale reliabilities using Raykov's (2009) unidimensional composite reliability measure.

Subsequently, we conducted multiple regression analyses. We entered gender as a covariate, given gender differences in narcissism (Grijalva et al., 2015b) and the shortage of female coaches that would allow for separate analyses based on gender. We opted for multiples regression as opposed to structural equation modeling, because of the relatively small sample size (Nicolas et al., 2011). To determine the significance of total, direct, and indirect (via empathic concern and dominance) effects of narcissism on controlling and autonomy‐supportive behaviors, we implemented Preacher and Hayes’ (2008) SPSS PROCESS macro. The regression model contained two mediators (empathic concern and dominance), and we tested the significance of specific indirect effects using bias‐corrected bootstrapped 95% confidence intervals with 5000 resamples (Preacher & Hayes, 2008). We standardized all variables before conducting mediation analyses; hence all direct effects are standardized effects. As recommended, we report 95% bias‐corrected CIs rather than *P* values (Preacher & Hayes, 2008).

## Results

### Preliminary analyses

First, we screened the data for multivariate outliers using Mahalanobis distance (*P* < 0.01; Tabachnik & Fidell, 2001). This statistic identified seven outliers, which we removed. Next, we screed the data for univariate outliers and, as a result, removed five further outliers (i.e., *z*‐score >3.29), resulting in a final sample of 211 coaches. We present, in Table 1, the correlations, composite reliability coefficients, means, and standard deviations for all study variables. All of them had high internal consistency and were normally distributed (skewness range: −0.97–1.10, kurtosis range: −0.42–1.67). Correlation coefficients ranged from small to moderate, and did not reveal any relations suggesting that multicollinearity (i.e., *r* > 0.70) could be an issue in subsequent regression analyses.

**Table 1:** Correlations, internal consistencies, means, and standard deviations for study variables (*N* = 211)

Variable	1	2	3	4	5
1 Narcissism	**.82**				
2 Dominance	.58**	**.83**			
3 Empathic concern	−.19**	−.20**	**.71**		
4 Controlling behaviors	.33**	.25**	−.23**	**.71**	
5 Autonomy‐supportive behaviors	−.07	−.06	.29**	−.28**	**.70**
Possible range	0–40	1–6	0–4	1–6	1–6
M	12.98	3.38	2.98	1.38	5.26
SD	5.79	0.83	0.63	0.40	0.51

Raykov composite reliability coefficients are in bold along the diagonal. Correlation values are below the diagonal.

*P < 0.05, **P < 0.01 (two‐tailed).

### Main analyses

**Figure 1:**
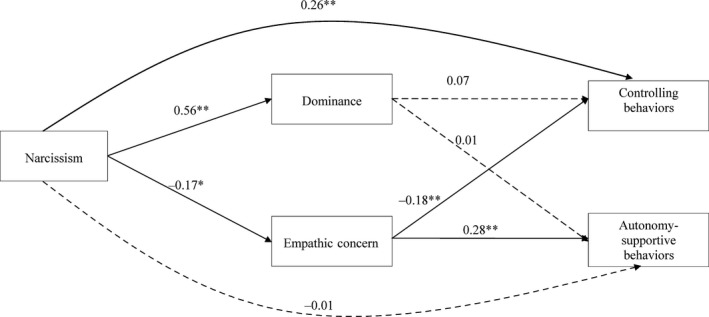
Testing the predicting effects of narcissism on controlling and autonomy‐supportive behaviours via empathic concern and dominance when controlling for gender (*N* = 211). *Note*: Standardised beta coefficients are reported. *P < 0.05, **P < 0.01. Dashed lines represent non‐significant relations.

To test the hypotheses, we conducted multiple regression analyses controlling for gender (Fig. 1).^1^ In the first model, we included narcissism as an independent variable, controlling behaviors as the outcome variable, and empathic concern and dominance as mediator variables. Narcissism positively predicted controlling behaviors (β = 0.26, *P* = 0.01), and negatively predicted empathic concern (β = −0.17, *P* = 0.01); empathic concern negatively predicted controlling behaviors (β = −0.18, *P* = 0.01). In addition, we obtained an indirect positive effect (Table 2) of narcissism on controlling behaviors via reduced empathic concern (*b* = 0.03; lower bound [LB] = 0.00; upper bound [UB] = 0.07). Ranges from LB to UB that do not include 0 are indicative of a true indirect effect (Preacher & Hayes, 2008). In contrast, although narcissism positively predicted dominance (β = 0.56, *P* < 0.01), there was no effect of dominance on controlling behaviors (β = 0.07, *P* = 0.38). Furthermore, there was no indirect effect of narcissism on controlling behaviors via dominance (*b* = 0.04; LB = −0.05; UB = 0.14).

**Table 2:** Total and indirect effects of narcissism on controlling and autonomy‐supportive behaviors via dominance and empathic concern when controlling for gender

Independent variable	Criterion variable	Total indirect effects (95% CI)	Specific indirect effects	
			Dominance (BC 95% CI)	Empathic Concern (BC 95% CI)
Narcissism	Controlling behaviors	.07 (−.02–.17)	.04 (−.05–.14)	.03 (.00–.07)
	Autonomy‐supportive behaviors	−.04 (−.13–.05)	.01 (−.08–.10)	−.05 (−.10–(−.01))

In the second model, we included narcissism as an independent variable, autonomy‐supportive behavior as the outcome variable, and empathic concern and dominance as mediator variables. Narcissism did not directly predict autonomy‐supportive behaviors (β = −0.01, *P* = 0.92). However, narcissism negatively predicted empathic concern (β = −0.17, *P* = 0.01), and empathic concern positively predicted autonomy‐supportive behaviors (β = 0.28, *P* < 0.01). Furthermore, there was an indirect negative effect (Table 2) of narcissism on autonomy‐supportive behaviors via empathic concern (*b* = −0.05; LB = −0.10; UB = −0.01). In contrast, although narcissism positively predicted dominance (β = 0.56, *P* < 0.01), there was no effect of dominance on autonomy‐supportive behaviors (β = 0.01, *P* = 0.87). Furthermore, there was no indirect effect of narcissism on autonomy‐supportive behaviors via dominance (*b* = 0.01; LB = −0.08; UB = 0.10).

## Discussion

We set out to investigate whether narcissism predicts controlling and autonomy‐supportive behaviors in situations in which narcissism could be activated, both directly and indirectly through empathic concern and dominance. We obtained partial support for the hypotheses in that narcissism positively predicted controlling behaviors, but the anticipated direct negative effect of narcissism on autonomy‐supportive behaviors did not emerge. Furthermore, empathic concern mediated the predictive effects of narcissism on both controlling and autonomy‐supportive behaviors as expected, but the hypothesized mediated effect of narcissism on controlling behaviors via dominance did not emerge.

The positive relation between narcissism and controlling behaviors is a novel finding in the SDT literature. This finding is consistent with the personality and social psychology literature, which has shown that narcissists engage in more control‐based behaviors (Nevicka et al., 2011), aggression (Bushman & Baumeister, 1998), and hostility (Raskin et al., 1991). There are several reasons why narcissistic coaches may utilize controlling behaviors, some of which we described in our Introduction. For example, when coaches feel that their superiority over their athletes is questioned, they may resort to controlling behaviors to bring their athletes “back in line,” as opposed to try and engage in a conversation with them or understand their perspective.

As expected, the effect of narcissism on coaches’ controlling behaviors was in part mediated by empathic concern. Coaches who were higher in narcissism experienced lower levels of empathy and, in turn, reported engaging in more controlling behaviors. According to the literature, narcissists’ lack of empathic concern is a spontaneous reaction driven by their opportunity to exploit subordinates (Hepper et al., 2014b; Schoel et al., 2015). Lack of empathy may be an explanation for why narcissistic coaches are unmotivated to try to understand their athletes’ feelings and resort in controlling behaviors (e.g., criticism, confrontation, yelling).

Contrary to our hypothesis, narcissism did not have a direct negative effect on autonomy‐supportive behaviors. As alluded to in the Introduction, whether narcissists will display autonomy‐supportive behaviors or not depends on the expected self‐enhancement benefits of such behavior. Unfortunately, we did not assess this potentially relevant moderator, and this omission might explain the null effects. Consistent with our hypotheses, narcissism had an indirect effect on autonomy‐supportive behaviors through empathic concern. Coaches who were higher in narcissism experienced lower levels of empathic concern and, in turn, had a lower likelihood of engaging in autonomy‐supportive behaviors. Empathy is a key motivator of prosocial behavior, as the ability to share and experience someone else's feelings increases the likelihood of helping (Eisenberg et al., 2010). Thus, non‐empathetic coaches may be less likely to engage in autonomy‐supportive behaviors, because they fail to appreciate how such prosocial acts will make athletes feel.

Contrary to our hypotheses, dominance did not mediate the effects of narcissism on controlling behaviors. Although the correlational pattern among narcissism, dominance behaviors, and controlling behaviors was consistent with a potential mediated effect, we detected no such effect in the regression analyses. An explanation for the disparity between the correlation and regression results could be that most of the effect on narcissism on controlling behaviors is direct and that dominance does not have unique predictive capacity over and above narcissism.

### Limitations and future directions

Our study has limitations. Given that it was based exclusively on coach self‐reports, it is possible that coaches’ responses were influenced by socially desirable responding. As such, future researchers may seek to replicate the findings by employing observational techniques (i.e., videotaping coach behaviors) or obtaining athlete perceptions of coach behaviors. Additionally, as we used a cross‐sectional design, we could not test causality. Future work would need to implicate quasi‐experimental designs. For example, one could ask participants, pre‐selected based on their narcissism scores (low vs high) to coach an athlete (confederate) in a laboratory task. Next, one would create situations such as those described in the scenarios used, and test whether such situations (e.g., entitlement) impact on the degree to which the narcissistic vs non‐narcissistic coach utilizes autonomy‐supportive and controlling behaviors in interacting with the athlete.

Another limitation concerns the sampling imbalance of male to female coaches. A recent meta‐analysis indicated that males are generally more narcissistic than females; however, the gender differences were small (Grijalva et al., 2015b). Our sample approximated the gender balance of the UK coach population: Mcllroy (2015) reported a much higher percentage of male (72%) than female (28%) coaches currently working in the United Kingdom. Nevertheless, future research could strive for more balanced coach recruitment based on gender.

Several additional research directions stem from our work. It would be interesting to explore the effect of coach narcissism on athletes’ self‐determined motivation and associated outcomes (Mageau & Vallerand, 2003). Based on findings that narcissistic leaders are often disliked by their followers (Judge et al., 2006; Schoel et al., 2015), it is possible that athletes coached by narcissists are less satisfied with their coach than athletes coached by non‐narcissists. Additionally, future research could consider athletes’ personality, as the dyadic relationship is likely to be influenced by athletes’ own narcissism (Wallace et al., 2015). Also, narcissism represents only one‐third of the Dark Triad (i.e., along with psychopathy and Machiavellianism; Paulhus & Williams, 2002). The Dark Triad factors share common characteristics such as self‐promotion, lack of empathy, and aggressiveness. Thus, psychopathy and Machiavellianism could also be explored as antecedents of coach interpersonal styles (Paulhus & Williams, 2002).

In summary, our findings extend the SDT literature by demonstrating that personality traits, such as narcissism, predict coaches’ likelihood of directly and indirectly utilizing controlling behaviors, and of indirectly utilizing autonomy support behaviors, in situations in which narcissism could be activated. As such, this study makes an important contribution to the SDT literature. The study identifies a key antecedent of coaching behaviors and improves understanding of potential explanatory mechanisms (i.e., empathetic concern) on how narcissism predicts behavior.

## Perspective

This research is, to the best of our knowledge, the first to examine the role of coach narcissism in sport. Our findings, in combination with much‐needed follow‐up investigations, could help sport psychology practitioners develop specific strategies for coaches in order to reduce the influence of narcissism on controlling behaviors and to promote autonomy‐supportive behaviors. Recent work supports the efficacy of interventions aimed at developing empathy in narcissistic populations (Hepper et al., 2014b). For example, investigations in educational settings have shown that empathic concern can be taught through interventions based on the development of peer‐facilitation skills (Hatcher et al., 1994) or via self‐affirmation techniques (e.g., writing about one's important values; Thomaes et al., 2009). Interventions such these may be generalizable to sport coaches.

## Supporting information


**Data S1.** Autonomy‐supportive and controlling behaviors measure of narcissistic coaches (scenarios).

## Note

1 We repeated the main analyses reported in the manuscript, separating maladaptive (i.e., exhibitionism, exploitativeness, entitlement) and adaptive (i.e., authority, self‐sufficiency) dimensions of narcissism (Barry & Malkin, 2010). Authority, exhibitionism, exploitativeness, and entitlement directly and positively predicted dominance. Entitlement directly and negatively predicted empathic concern. Dominance was not a direct significant predictor of autonomy‐supportive and controlling behaviors. Empathic concern was a direct negative predictor of controlling behaviors, and a direct positive predictor of autonomy‐supportive behaviors. When examining indirect effects, empathic concern was a significant mediator between entitlement and controlling and autonomy‐supportive behaviors, respectively.
