# Supplementary material for: Narcissism and coach interpersonal style: A self‐determination theory perspective
Source: Scand J Med Sci Sports. 2015 Dec 21;27(2):254–61. doi: 10.1111/sms.12635 (PMC6849559; doi:10.1111/sms.12635)
Supplement: Supplementary file 1 — Data S1. Autonomy‐supportive and controlling behaviors measure of narcissistic coaches (scenarios). [file SMS-27-254-s001.docx]

**Narcissism and Coach Behaviours: A Self-Determination Theory Perspective
Autonomy-Supportive and Controlling Behaviours Measure of Narcissistic Coaches (Scenarios)**

These items pertain to a series of hypothetical scenarios. Each scenario describes a sport specific situation involving a coach and two ways that the coach could respond to this situation. All the scenarios refer to male coaches; however they are equally applicable to female coaches. Also, scenarios are described in a team sport setting, but could be applicable to the individual sport setting as well. Please ignore the gender of the coach and the sport setting while reading the scenarios below and focus on the behaviours only. Please read each scenario and the two possible coach responses. Then indicate how much you personally agree or disagree with each possible response. **Think of each response option in terms of what you think would be most appropriate for a coach of the level you are coaching in to do in the given situation.**

ANSWER SCALE:

1 2 3 4 5 6

STRONGLY STRONGLY

DISAGREE AGREE

**Scenario 1 (Hypersensitivity to criticism)**

Upon the end of an important league game, the coach gathered his team on the field to discuss the team’s defeat. After the coach finished talking, a team captain stood up criticising the coach for the way the team played. The coach was visibly insulted and became intensely hostile in response to the criticism.

*What would be appropriate for a coach to do in this situation?*

1. Shout at the player, threatening his captain position. **(controlling)**

2. Invite the player to a one-on -one meeting, to discuss how things might be resolved. **(autonomy-supportive)**

**Scenario 2 (Authority)**

During the training session today, one of the players violated one of the rules of conduct established by the coach. The player apologized to the coach, but the player’s apology was deemed unacceptable by the coach. This coach has a very strict and inflexible policy on failing to obey established rules.

*What would be appropriate for a coach to do in this situation?*

1. Show to the player his disappointment by paying less or no attention to the player. **(controlling)**

2. Accept the apology that day, but still verbally remind the player right after the session
 about the importance of following established rules. **(autonomy-supportive)**

**Scenario 3 (Self-sufficiency)**

Two of the players got frustrated about their coach always making all the game decisions without accepting any advice from assistant coaches and players, because the coach doesn’t think he needs them. Both of the players were considering transferring to another team.

*What would be appropriate for a coach to do in this situation?*

1. Give players a chance to talk to him and explain alternative ways of them being involved
 in a game decision. **(autonomy-supportive)**

2. Tell the players he is the boss on the team and they are free to transfer to another team, if
 they cannot accept that kind of regime. **(controlling)**

**Scenario 4 (Superiority)**

A player was in the process of registering to play for the team next year. The player wanted to stay on the same team, but was annoyed with the coach always bragging about how special he (the coach) is and how he is so much better than other coaches.

*What would be appropriate for a coach to do in this situation to keep the player on the team?*

1. Allow the player to share his thoughts and answer any questions fully and carefully. **(autonomy-supportive)**

2. Tell the player that he would let down everyone on the team down if he left. **(controlling)**

**Scenario 5 (Exhibitionism)**

This coach wants to be in the centre of attention. He is always showing off by demonstrating how good he is with certain game components (e.g., taking a penalty). Some of the players were rude to him trying to make him stop doing that.

*What would be appropriate for a coach to do in this situation?*

1. The next training session punish those players with an extra work out for unacceptable behaviour. **(controlling)**

2. Respect that different people have different views on this matter and say so to his players. **(autonomy-supportive)**

**Scenario 6 (Exploitativeness)**

Despite the fact that the player was seriously injured all season long, the coach was still playing him in every game throughout the season. The team won the final game, although the player’s injury got worse afterwards. The coach was very happy about winning the title, but the player was miserable and in pain.

*What would be appropriate for a coach to do in this situation?*

1. Talk to the player, acknowledge how the player feels and offer advice regarding rehabilitation. **(autonomy-supportive)**

2. Tell player to “man up” and deal with it. Injuries are part of sport. **(controlling)**

**Scenario 7 (Entitlement)**

A key player was told by his coach that it was crucial for him to perform above average. The coach really wanted his team to win the title, because he thought he fully deserved it after years of trying. The player played below coach’s expectations and the team lost the final crucial game.

*What would be appropriate for a coach to do in this situation?*

1. Let the player know in front of the whole team that he let the coach down. **(controlling)**

2. Talk it over with the player to understand further what the problem was. **(autonomy-supportive)**

**Scenario 8 (Feelings of Inferiority)**

A coach always appears to try hard to be confident, if not arrogant, about his coaching abilities. However, every time during games played against an opponent of similar or better ranking, he appears pessimistic when talking to his players, saying things such as “it’s a difficult game, we may be beaten” and “I hope we do not fail”, giving the impression that he is unsure about himself. In the last game, one of the players remarked that the coach might have a problem coaching effectively against equal or better opposition.

*What would be appropriate for a coach to do in this situation?*

1. Provide rationale to the player why he is acting the way he is during the game time. **(autonomy-supportive)**

2. Discipline the player via a fine or other form of punishment. **(controlling)**

**Scenario 9 (Lack of Empathy)**

Following death in his family, a player came back to training. The player was feeling run-down emotionally, and his performance in training was weak. The coach was unhappy about the player’s performance and showed no understanding of his personal circumstances. The player went home and did not return the next day.

*What would be appropriate for a coach to do in this situation?*

1. Confront the player and question his loyalty to the team. **(controlling)**

2. Invite the player for a discussion to get his perspective. **(autonomy-supportive)**

**Scenario 10 (Amorality)**

In the last game, a player failed to listen to the coach’s instructions about discretely hurting an opponent in order to gain advantage and score points.

*What would be appropriate for a coach to do in this situation?*

1. Leave the player in the game, but remind him that he needs to be listening to the coach’s

instructions and that, if he has any disagreements, he should express them ahead of time.
 **(autonomy-supportive)**

2. Promise the player a reward, if he does as told. **(controlling)**

**Scenario 11 (Arrogance)**

During the game the coach was berating an official for what he considered an unfair decision. The coach considered he knew the rules of the sport better than the official. One of the players tried to calm the coach down by explaining to him it was a fair decision and he was not right this time.

*What would be appropriate for a coach to do in this situation?*

1. Explain to the player the reason why he acted like that. **(autonomy-supportive)**

2. Shout at the player in front of others and make the player apologise. **(controlling)**

**Scenario 12 (Grandiosity)**

At the sports banquet, the coach was supposed to give a speech on the achievements of his team. However, he spent most of the time highlighting the successes in the first person, as if he was the only one responsible for them. When it was the turn for the best athlete to talk, the player praised everyone on the team for the accomplishments and said a simple “thank you” to the coach. The coach was furious for not receiving more credit from the player.

*What would be appropriate for coach to do in the situation?*

1. Not applaud him after his speech and visibly show his disappointment. **(controlling)**

2. Applaud the player with the rest of his teammates and praise him for being a team player. **(autonomy-supportive)**

NOTE:

All the answers were scored in the same direction (no reverse coding). The higher score on the scale represents higher autonomy-supportive and controlling behaviours, respectively.

Narcissistic personality traits and behaviours (i.e., autonomy-supportive, controlling) described in the scenario and responses are named in the brackets.
